# Prevalence and Predictors of Respiratory Diseases Among Coal-Based Sponge Iron Plant Workers: A Cross-Sectional Study in Barjora, India

**DOI:** 10.5334/aogh.2424

**Published:** 2019-01-22

**Authors:** Kaushik Chattopadhyay

**Affiliations:** 1 Epidemiology and Population Health, London School of Hygiene and Tropical Medicine, Keppel Street, London, UK

## Abstract

**Background:**

During the last decade, coal-based sponge iron plants, a highly polluted industry, have grown rapidly in Barjora, India. The toxic effects of particulate matters and gaseous pollutants are often rapid and include respiratory diseases (such as asthma and rhinoconjunctivitis).

**Objectives and Methods:**

A cross-sectional study was conducted among 258 coal-based sponge iron plant workers in Barjora to assess the prevalence of respiratory diseases (self-reported) and to determine the associated factors.

**Findings:**

The percentage of participants with any chronic respiratory disease, asthma and rhinoconjuctivitis were 25.5%, 8.9%, and 17.1%, respectively. The odds of any chronic respiratory disease were lower in participants with family history of any chronic respiratory disease (odds ratio [OR] 0.47, 95% confidence interval [CI] 0.24–0.91, *P* = 0.024). The odds of asthma were lower in participants living in a room with less than 3 people (OR 2.86, 95% CI 1.16–7.07, *P* = 0.023) and with family history (OR 0.20, 95% CI 0.08–0.53, *P* = 0.001). The odds of rhinoconjuctivitis were lower in illiterate participants (OR 0.34, 95% CI 0.12–0.94, *P* = 0.038) and those with pucca/semipucca house type (OR 2.44, 95% CI 1.11–5.39, *P* = 0.027).

**Conclusion:**

Many coal-based sponge iron plant workers in Barjora report the presence of respiratory diseases, and the predictors such as overcrowding and poor quality housing were identified.

## Introduction

Sponge iron or direct-reduced iron (DRI) is a transitional material used in the production of steel. Either coal or natural gas is used in sponge iron production. In India, non coking coal is easily available. Thus, the sector depends mostly on coal-based sponge iron and nearly 80% of the total coal-based sponge iron plants are located in India [1]. About 60% of this production comes from small-scale industries in the unorganized sector with poor pollution control facilities [1]. During the past decade, these sponge iron plants have rapidly grown in the Barjora block of Bankura district, a deprived district in West Bengal [2]. These factories are categorized as red industries (highly polluted industries), and the major pollutants are of three types: solid waste heavy metals (cadmium, chromium, lead, mercury, and nickel); particulate matters (suspended particulate matter and respirable particulate matter); and gaseous pollutants (hydrocarbons and oxides of sulfur and nitrogen,) [1,2,3,4]. The toxic effects of solid waste heavy metals are varied and often take several years to manifest. However, the toxic effects of particulate matters and gaseous pollutants are often rapid and include respiratory diseases (such as asthma and rhinoconjunctivitis) [1,2,3,4]. The presence of respiratory diseases places a burden on the individual, family, community, and health services. However, no research has been conducted to assess the prevalence of respiratory diseases among coal-based sponge iron plant workers. The aim of the study was to assess the prevalence of respiratory diseases among coal-based sponge iron plant workers and to determine the associated factors. Knowledge of these associated factors would provide valuable information about strategies that professionals and providers of health care can address to manage respiratory diseases among coal-based sponge iron plant workers.

## Methods

### Study Design, Participants, Area, and Inclusion/Exclusion Criteria

**Figure 1:**
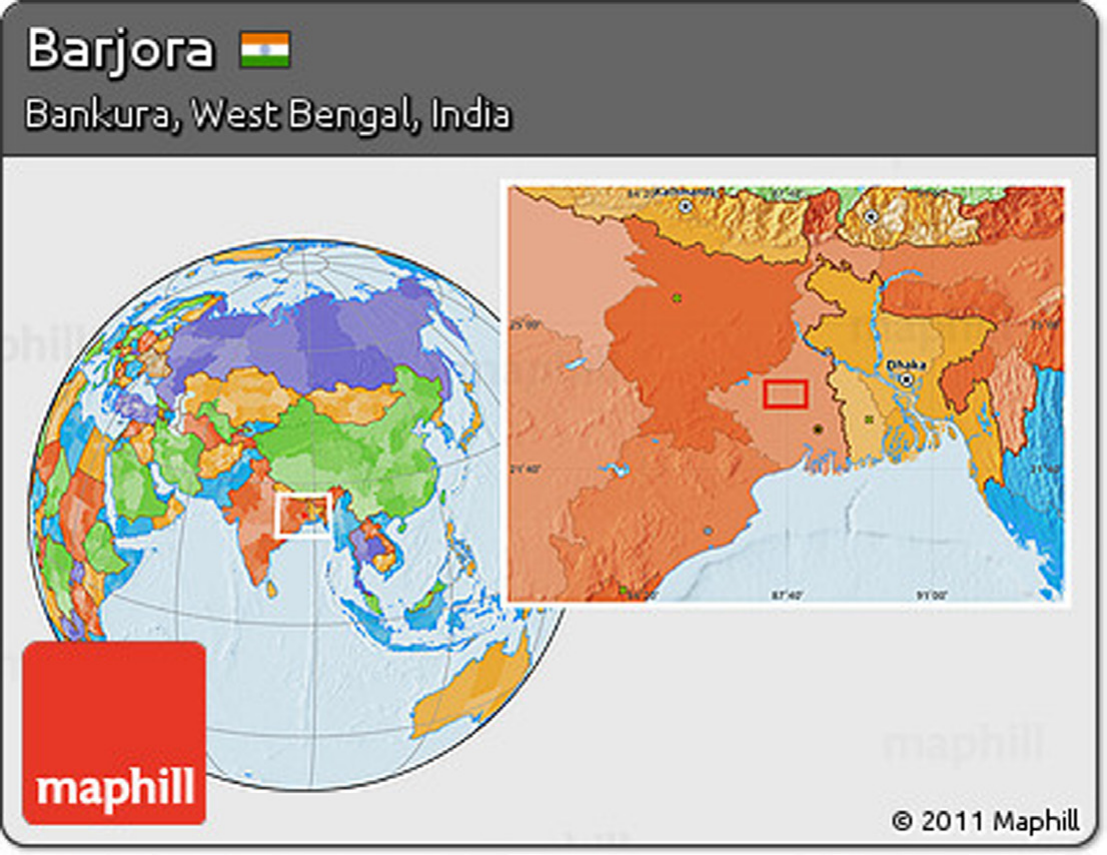
Map of Barjora, West Bengal, India.

A cross-sectional study was conducted among coal-based sponge iron plant workers in Barjora block (Bankura district, West Bengal, India) as shown in Figure 1. Participants who gave written informed consent to participate in the study were included, and those who were absent from work on the dates of the survey were excluded.

### Data Collection Procedure and Tool

A survey was conducted with a quantitative questionnaire (available in English, Bengali, and Hindi languages) in May and June 2013. The questionnaire was either self-completed by the participant (if literate) or was completed by the field worker (for illiterate participants). In the latter case, each question was shown and read to the participant who was asked to say and point out the answer. Each session was completed in one sitting. The data were entered on the day of its collection.

The questionnaire included the following sociodemographic and occupational variables: age (in years), sex, mother tongue (proxy measures for local ethnic origin, culture, and lifestyle), religion, social caste (general or scheduled caste [SC]/scheduled tribe [ST]/other backward class [OBC]), marriage, education (literate or illiterate), work type (nonmanual or manual), working hours per day (8 [normal] [5] or more than 8), salary per month (5395 Indian rupees [INR; minimum monthly wage of an unskilled worker] [5] or more, or less than 5395 INR), total duration of work in this type of factory (proxy measure for exposure time, in years), smoking, smokeless tobacco intake, alcohol drinking, house type (pucca/semipucca [at least some high-quality construction materials such as bricks, tiles, cement, and concrete] or kachcha [low-quality construction materials such as mud and thatch]) [6], people living in a room (less than 3, or 3 or more to indicate crowding) [6], pet animal at home, domestic cooking fuel (cleaner fuel [noncontinuous exposure to smoke: charcoal, coal/coke/lignite, kerosene, electricity, petroleum gas, and biogas] or biomass fuel [continuous exposure to smoke: wood/crop-residues and animal dung]) [6], separate room as kitchen at home, chimney/exhaust fan for cooking food at home, family history (presence of any chronic respiratory disease in biological father/mother/siblings), and health insurance. The presence of any respiratory disease was probed with the following question: Do you have any chronic respiratory disease, asthma or rhinoconjunctivitis, diagnosed by a medical doctor? The hint provided for the word *asthma* was symptoms like coughing, wheezing, chest tightness, and breathlessness [7]. The hint provided for the word *rhinoconjunctivitis* was symptoms like nasal congestion, runny nose, postnasal drip, sneezing, red eyes (conjunctivitis), and itching of the nose or eyes [8]. All the variables were dichotomous except age and total duration of work in this type of factory. The questionnaire was designed in English, translated into Bengali and Hindi, reviewed by a local primary school teacher, and pretested extensively on 6 local similar workers who were not involved in the study.

### Sample Size

Because this was the first study on the prevalence of respiratory diseases among coal-based sponge iron plant workers, no information was available on which to base the sample size calculation. Instead, a web-based sample size calculator, Creative Research Systems [9], was used to calculate the sample size, using the following assumptions/information: confidence level (95%), margin of error (5%) and population size (662 information from the factory directors in Barjora). A random sample of 258 such workers was required, assuming a response rate of 95%. A numbered list of all 662 workers was created (worker #1, worker #2, worker #3, and so forth). A web-based randomizer, Research Randomizer [10], was used to generate 1 set of 258 unique, sorted numbers with a range from 1 to 662 (representing the workers’ assigned numbers).

### Ethics

Approval was received from the Barjora Block Development Office Committee (the committee was based on the Indian Council of Medical Research Ethical Guidelines for Biomedical Research on Human Participants, 2006) [11]. Information sheets and consent forms were available in English, Bengali, and Hindi. The study objectives were explained to all the eligible participants and written informed consent was taken from those interested in participating. Illiterate participants were requested to put their left-hand thumb impression on the consent form. Participants were not compelled and were free to participate in the study. They were assured regarding the anonymity, privacy, confidentiality, and data protection of their information.

### Statistical Analyses

The presence of any chronic respiratory disease, asthma, or rhinoconjunctivitis was categorized into *no* and *yes*, and numbers and proportions were calculated being a categorical variable. Appropriate methods were used to investigate the association between the presence of any chronic respiratory disease, asthma, or rhinoconjunctivitis, and sociodemographic and occupational variables (χ^2^ test or simple logistic regression). To identify any independent association, corresponding multiple logistic regression models were developed using the backward stepwise regression analysis and all the sociodemographic and occupational variables were included. Sensitivity analyses were carried out, and only those variables with *P* value of 0.20 in the univariate analyses were included in the multiple regression models. Multiple regression models included a sample with unknown values for these adjusted variables. Odds ratios (ORs) and their respective 95% confidence intervals (CI) were calculated. The results were considered significant when *P* values were ≤0.05. All data were analyzed using Stata Statistical Software Version 12 for Windows software [12].

## Results

The response rate was 100%. All the participants were men with a mean age of 35.4 years. The percentage of participants with any chronic respiratory disease, asthma, and rhinoconjunctivitis were 25.5%, 8.9%, and 17.1%, respectively. Table 1 reports the characteristics of coal-based sponge-iron factory workers with and without any chronic respiratory disease, asthma, or rhinoconjunctivitis. Family history (*P* = 0.017) was found to be associated with the presence of any chronic respiratory disease. People living in a room (*P* = 0.024) and family history (*P* < 0.001) were found to be associated with the presence of asthma. Table 2 shows the multiple backward stepwise regression analyses to determine factors independently associated with the presence of any chronic respiratory disease, asthma, or rhinoconjunctivitis. The odds of any chronic respiratory disease were lower in participants with family history (OR 0.47, 95% CI 0.24–0.91, *P* = 0.024). The odds of asthma were lower in participants living in a room with <3 people (OR 2.86, 95% CI 1.16–7.07, *P* = 0.023) and with family history (OR 0.20, 95% CI 0.08–0.53, *P* = 0.001). The odds of rhinoconjunctivitis were lower in illiterate participants (OR 0.34, 95% CI 0.12–0.94, 0.038) and those with pucca/semipucca house type (OR 2.44, 95% CI 1.11–5.39, *P* = 0.027). Sensitivity analyses showed similar results (i.e., the results were significant) except the association between the presence of rhinoconjunctivitis and education.

**Table 1:** Characteristics of Coal-based Sponge Iron Factory Workers With and Without Any Chronic Respiratory Disease, Asthma, or Rhinoconjunctivitis.

	Any chronic respiratory disease (n = 258, includes 11 unknown)						Asthma (n = 258)						Rhinoconjunctivitis (n = 258)					

	No (n = 181)		Yes (n = 66)		*P*		No (n = 235)		Yes (n = 23)		*P*		No (n = 214)		Yes (n = 44)		*P*	

Age	35.5 (8.5)	*	35.0 (7.4)	*	0.710	^†^	35.5 (8.2)	*	34.5 (7.7)	*	0.582	^†^	35.8 (8.3)	*	33.5 (7.3)	*	0.085	^†^
Mother tongue					0.803						0.490						0.287	
Bengali	155 (85.6)		58 (87.9)				200 (85.1)		22 (95.7)				186 (86.9)		36 (81.8)			
Other	21 (11.6)		7 (10.6)				28 (11.9)		1 (4.3)				22 (10.3)		7 (15.9)			
Unknown	5 (2.8)		1 (1.5)				7 (3.0)		0				6 (2.8)		1 (2.3)			
Religion					1.000						0.256						0.406	
Hindu	173 (95.6)		63 (95.5)				226 (96.2)		21 (91.3)				206 (96.3)		41 (93.2)			
Islam	8 (4.4)		3 (4.5)				9 (3.8)		2 (8.7)				8 (3.7)		3 (6.8)			
Social caste					0.195						0.354						0.458	
General	109 (60.2)		45 (68.2)				145 (61.7)		16 (69.6)				137 (64.0)		24 (54.6)			
SC/ST/OBC	69 (38.1)		19 (28.8)				86 (36.6)		6 (26.1)				75 (35.1)		17 (38.6)			
Unknown	3 (1.7)		2 (3.0)				4 (1.7)		1 (4.3)				2 (0.9)		3 (6.8)			
Marriage					0.638						0.196						0.150	
Yes	157 (86.7)		54 (81.8)				204 (86.8)		17 (73.9)				186 (86.9)		35 (79.6)			
No	24 (13.3)		10 (15.2)				30 (12.8)		5 (21.7)				26 (12.2)		9 (20.4)			
Unknown	0		2 (3.0)				1 (0.4)		1 (4.4)				2 (0.9)		0			
Education					0.568						1.000						0.171	
Literate	156 (86.2)		59 (89.4)				205 (87.2)		21 (91.3)				190 (88.8)		36 (81.8)			
Illiterate	24 (13.3)		7 (10.6)				29 (12.4)		2 (8.7)				23 (10.7)		8 (18.2)			
Unknown	1 (0.5)		0				1 (0.4)		0				1 (0.5)		0			
Work type					0.239						0.318						0.372	
Nonmanual	34 (18.8)		17 (25.8)				50 (21.3)		7 (30.4)				45 (21.0)		12 (27.3)			
Manual	146 (80.7)		49 (74.2)				184 (78.3)		16 (69.6)				168 (78.5)		32 (72.7)			
Unknown	1 (0.5)		0				1 (0.4)		0				1 (0.5)		0			
Hours/day					0.452						1.000						1.000	
8	161 (89.0)		62 (93.9)				212 (90.2)		21 (91.3)				193 (90.2)		40 (90.9)			
>8	18 (9.9)		4 (6.1)				21 (8.9)		2 (8.7)				19 (8.9)		4 (9.1)			
Unknown	2 (1.1)		0				2 (0.9)		0				2 (0.9)		0			
Salary/month					0.886						0.975						0.367	
≥5395 INR	45 (24.9)		17 (25.8)				62 (26.4)		6 (26.1)				54 (25.2)		14 (31.8)			
<5395 INR	136 (75.1)		49 (74.2)				173 (73.6)		17 (73.9)				160 (74.8)		30 (68.2)			
Total duration	9.9 (13.7)	*	9.4 (11.6)	*	0.667	^†^	10.2 (14.7)	*	7.9 (3.1)	*	0.974	^†^	10.5 (15.3)	*	7.7 (3.3)	*	0.557	^†^
Smoking					0.541						0.438						0.373	
No	106 (58.6)		36 (54.5)				133 (56.6)		15 (65.2)				120 (56.1)		28 (63.6)			
Yes	74 (40.9)		30 (45.5)				101 (43.0)		8 (34.8)				93 (43.4)		16 (36.4)			
Unknown	1 (0.5)		0				1 (0.4)		0				1 (0.5)		0			
Smokeless tobacco					0.444						0.659						0.755	
No	89 (49.2)		29 (43.9)				113 (48.1)		10 (43.5)				101 (47.2)		22 (50.0)			
Yes	91 (50.3)		37 (56.1)				121 (51.5)		13 (56.5)				112 (52.3)		22 (50.0)			
Unknown	1 (0.5)		0				1 (0.4)		0				1 (0.5)		0			
Drinking					0.086						0.809						0.081	
No	119 (65.8)		36 (54.6)				146 (62.1)		15 (65.2)				139 (64.9)		22 (50.0)			
Yes	60 (33.1)		30 (45.4)				87 (37.0)		8 (34.8)				74 (34.6)		21 (47.7)			
Unknown	2 (1.1)		0				2 (0.9)		0				1 (0.5)		1 (2.3)			
House type					0.925						0.858						0.220	
Pucca/semipucca	88 (48.6)		32 (48.5)				116 (49.3)		11 (47.8)				102 (47.6)		25 (56.8)			
Kachha	91 (50.3)		34 (51.5)				117 (49.8)		12 (52.2)				111 (51.9)		18 (40.9)			
Unknown	2 (1.1)		0				2 (0.9)		0				1 (0.5)		1 (2.3)			
People in a room					0.115						0.024						0.672	
<3	63 (34.8)		16 (24.2)				69 (29.4)		12 (52.2)				66 (30.8)		15 (34.1)			
≥3	118 (65.2)		50 (75.8)				166 (70.6)		11 (47.8)				148 (69.2)		29 (65.9)			
Pet					0.564						0.390						0.503	
No	83 (45.9)		33 (50.0)				114 (48.5)		9 (39.1)				100 (46.7)		23 (52.3)			
Yes	98 (54.1)		33 (50.0)				121 (51.5)		14 (60.9)				114 (53.3)		21 (47.7)			
Domestic cooking fuel					0.303						0.213						0.791	
Cleaner fuel	107 (59.1)		44 (66.7)				142 (60.4)		17 (73.9)				131 (61.2)		28 (63.6)			
Biomass fuel	73 (40.3)		22 (33.3)				92 (39.2)		6 (26.1)				82 (38.3)		16 (36.4)			
Other	1 (0.6)		0				1 (0.4)		0				1 (0.5)		0			
Kitchen					0.786						0.998						0.579	
Yes	134 (74.0)		48 (72.7)				173 (73.6)		17 (73.9)				156 (72.9)		34 (77.3)			
No	46 (25.4)		18 (27.3)				61 (26.0)		6 (26.1)				57 (26.6)		10 (22.7)			
Unknown	1 (0.6)		0				1 (0.4)		0				1 (0.5)		0			
Chimney/exhaust fan					0.482						0.430						0.190	
Yes	14 (7.7)		7 (10.6)				19 (8.1)		3 (13.0)				16 (7.5)		6 (13.6)			
No	166 (91.7)		59 (89.4)				214 (91.0)		20 (87.0)				196 (91.6)		38 (86.4)			
Unknown	1 (0.6)		0				2 (0.9)		0				2 (0.9)		0			
Family history					0.017						0.001						0.654	
No	137 (75.7)		40 (60.6)				173 (73.6)		9 (39.1)				151 (70.6)		31 (70.5)			
Yes	33 (18.2)		21 (31.8)				45 (19.2)		11 (47.8)				45 (21.0)		11 (25.0)			
Unknown	11 (6.1)		5 (7.6)				17 (7.2)		3 (13.1)				18 (8.4)		2 (4.5)			
Health insurance					0.451						0.584						0.880	
No	35 (19.3)		10 (15.1)				46 (19.6)		3 (13.0)				41 (19.2)		8 (18.2)			
Yes	146 (80.7)		56 (84.9)				189 (80.4)		20 (87.0)				173 (80.8)		36 (81.8)			

n (%), χ^2^ test. *P* value excludes unknown. INR, Indian rupees; OBC, other backward class; SC, scheduled caste; ST, scheduled tribe. * Mean (SD). ^†^ Simple logistic regression.

**Table 2:** Multiple Backward Stepwise Regression Analyses to Determine Factors Independently Associated with the Presence of any Chronic Respiratory Disease, Asthma, or Rhinoconjunctivitis.

	OR	95% CI	*P*

**Any chronic respiratory disease**			
Family history			
No	1		0.024
Yes	0.47	0.24–0.91	
**Asthma**			
People in a room			
<3	1		0.023
≥3	2.86	1.16–7.07	
Family history			
No	1		0.001
Yes	0.20	0.08–0.53	
**Rhinoconjunctivitis**			
Age	1.04	1.00–1.09	0.078
Education			
Literate	1		0.038
Illiterate	0.34	0.12–0.94	
Drinking			
No	1		0.096
Yes	0.56	0.28–1.11	
Social caste			
General	1		0.517
SC/ST/OBC	0.77	0.36–1.68	
House type			
Pucca/semipucca	1		0.027
Kachha	2.44	1.11–5.39	

OBC, other backward class; SC, scheduled caste; ST, scheduled tribe.

## Discussion

The percentage of participants with any chronic respiratory disease, asthma, and rhinoconjunctivitis were 25.5%, 8.9%, and 17.1%, respectively. These findings are consistent with other studies conducted in different occupational settings where smoke and dust are common [13,14,15,16,17,18]. The odds of asthma were lower in participants living in a room with <3 people. This is consistent with other studies, and overcrowding is known to be a risk factor for asthma [19,20,21]. Overcrowding is common in Indian population and may be viewed as a marker for poor indoor hygiene and consequently increased exposure to various indoor allergens (such as house dust mites, animal dander, cockroaches, fungi, and molds) that are known to play an important role in the pathogenesis of asthma [19,20]. The odds of rhinoconjunctivitis were lower in participants with pucca/semipucca house type. This is consistent with other studies, and poor quality housing is known to be a risk factor for rhinoconjuctivitis [19,22,23]. The odds of rhinoconjunctivitis were lower in illiterate participants. Intuitively, one would expect illiterates to have more rhinoconjunctivitis than literates. In this study, more illiterate participants may have reported the absence of rhinoconjunctivitis to field workers (who completed the questionnaire on their behalf) compared with self-completion by literate participants. In some studies, participants reported better health status during face-to-face interviews than in postal surveys [24,25]. This issue requires further exploration. The odds of any chronic respiratory disease or asthma were lower in participants with family history. Intuitively, one would expect the opposite and this issue requires further investigation.

This study has a number of strengths and weaknesses. To the best of the author’s knowledge, this is the first study on the prevalence of respiratory diseases among coal-based sponge iron plant workers. All the workers who were approached to participate in the study responded (a 100% response rate and thus no nonrespondents). This indicates that the data collection methodology was appropriate and there is more certainty in the study findings (ie, it is more likely the results are representative of the population). In terms of generalizability, the study findings could be valid in settings with similar populations and health care systems (such as in other South Asian countries). The standard steps in questionnaire development (design, translation, and pretesting) were followed to ensure the validity and reliability of the questionnaire. The field workers used a standardized protocol for data collection. The field workers and the participants belonged to the same culture, which minimized the scope for cultural bias in the study. Missing data could lead to bias, but it was extremely low in this study. Multiple regression analyses included a sample with missing values for the adjusted variables. Participants who were absent from work on the dates of the survey were excluded, and this absence from work could be due to respiratory diseases, which could have underestimated the prevalence of respiratory diseases. Most of the data were self-reported, and recall error could have been a problem. Medical records might be a more reliable measure (for the presence of any respiratory disease), but these were not available or accessible in the study area. The study focused on confirmed cases and excluded undiagnosed cases, which could have underestimated the prevalence of respiratory diseases. Because of limited resources and budget, lung function or other diagnostic tests could not be performed. However, these tests could be used in future studies, which would cross-check these study findings and would provide a complete picture of the scenario. Some of the associations that were found in the study deserve further examination. For example, the odds of any chronic respiratory disease or asthma were lower in participants with family history. It is possible that these findings were the result of other confounding factors not adjusted for in the models. The aim of the study was to explore the prevalence of respiratory diseases among coal-based sponge iron plant workers, and there was no control group in the study. The study findings are compared with other studies conducted in different occupational settings where smoke and dust are common [13,14,15,16,17,18], because similar studies conducted among coal-based sponge iron plant workers are lacking. Thus, similar research needs to be conducted among coal-based sponge iron plant employees working in other parts of India and other countries to enhance the generalizability of these results. Because this was a cross-sectional study, it was not possible to determine the causal association between different variables and the prevalence of respiratory diseases. A long-term, longitudinal study should be conducted among these coal-based sponge iron factory workers to assess the impact of various factors (these as well as other potential factors) on the prevalence of respiratory diseases. A good example would be to have a cohort study comparing coal-based sponge iron factory workers with other types of factory workers (healthy worker effect), rather than with the general population.

## Conclusions

Many coal-based sponge iron plant workers in Barjora report the presence of respiratory diseases, and the predictors, such as overcrowding and poor quality housing, were identified. The study findings could be taken into consideration in future interventional studies aimed at managing respiratory diseases of these workers.
